# Supplementary material for: GA4GH Phenopackets: A Practical Introduction
Source: Adv Genet (Hoboken). 2022 Aug 25;4(1):2200016. doi: 10.1002/ggn2.202200016 (PMC10000265; doi:10.1002/ggn2.202200016)
Supplement: Supplementary file 1 — Supporting Information [file GGN2-4-2200016-s005.pdf]

ADVANCED GENETICS
Open Access

Supporting Information

for *Advanced Genetics*, DOI 10.1002/ggn2.202200016

GA4GH Phenopackets: A Practical Introduction

*Markus S. Ladewig, Julius O. B. Jacobsen, Alex H. Wagner, Daniel Danis, Baha El Kassaby, Michael Gargano, Tudor Groza, Michael Baudis, Robin Steinhaus, Dominik Seelow, Nikolaos E. Bechrakis, Christopher J. Mungall, Paul N. Schofield, Olivier Elemento, Lindsay Smith, Julie A. McMurry, Monica Munoz-Torres, Melissa A. Haendel and Peter N. Robinson**

```yaml
family:
  id: "family.1"
  proband:
    id: "phenopacket.id.1"
    subject:
      id: "son.1"
      timeAtLastEncounter:
        age:
          iso8601duration: "P10Y2M4D"
      sex: "MALE"
    phenotypicFeatures:
    - type:
        id: "HP:0000407"
        label: "Sensorineural hearing impairment "
      onset:
        ontologyClass:
          id: "HP:0003577"
          label: "Congenital onset"
    metaData:
      created: "2022-04-17T10:35:00Z"
      createdBy: "biocurator"
      resources:
      - id: "hp"
        name: "human phenotype ontology"
        url: "http://purl.obolibrary.org/obo/hp.owl"
        version: "2022-04-15"
        namespacePrefix: "HP"
        iriPrefix: "http://purl.obolibrary.org/obo/HP_"
      phenopacketSchemaVersion: "2.0"
  pedigree:
    persons:
    - familyId: "family.1"
      individualId: "father.1"
      paternalId: "0"
      maternalId: "0"
      sex: "MALE"
      affectedStatus: "UNAFFECTED"
    - familyId: "family.1"
      individualId: "mother.1"
      paternalId: "0"
      maternalId: "0"
      sex: "FEMALE"
      affectedStatus: "UNAFFECTED"
    - familyId: "family.1"
      individualId: "daughter.1"
      paternalId: "father.1"
      maternalId: "mother.1"
```

```yaml
    sex: "FEMALE"
    affectedStatus: "UNAFFECTED"
  - familyId: "family.1"
    individualId: "son.1"
    paternalId: "father.1"
    maternalId: "mother.1"
    sex: "MALE"
    affectedStatus: "AFFECTED"
  - familyId: "family.1"
    individualId: "daughter.2"
    paternalId: "father.1"
    maternalId: "mother.1"
    sex: "FEMALE"
    affectedStatus: "UNAFFECTED"
files:
- uri: "/data/samples/vcf/example_000001215.vcf.gz"
  individualToFileIdentifiers:
    father.1: "sample.1"
    mother.1: "sample.2"
    daughter.1: "sample.3"
    son.1: "sample.4"
    daughter.2: "sample.5"
  fileAttributes:
    genomeAssembly: "GRCh38"
    fileFormat: "VCF"
    description: "multi-sample VCF file for family.1"
metaData:
  created: "2022-04-17T10:35:00Z"
  createdBy: "biocurator"
  phenopacketSchemaVersion: "2.0"
```
